# Performance of disc diffusion and gradient strip test on different Mueller–Hinton agar media plates and ComASP® and Bruker UMIC^®^ Cefiderocol Microdilution Panels for cefiderocol susceptibility testing in carbapenem-resistant *Pseudomonas aeruginosa*

**DOI:** 10.1093/jac/dkaf301

**Published:** 2025-08-18

**Authors:** Stefano Mancini, Helena M B Seth-Smith, Natalia Kolesnik-Goldmann, Melanie Mermod, Vladimira Hinic, Tim Roloff, Muhammad Ali Syed, Irfan Ullah, Adrian Egli, Oliver Nolte

**Affiliations:** Institute of Medical Microbiology, University of Zurich, Zurich, Switzerland; Institute of Medical Microbiology, University of Zurich, Zurich, Switzerland; Institute of Medical Microbiology, University of Zurich, Zurich, Switzerland; Institute of Medical Microbiology, University of Zurich, Zurich, Switzerland; Institute of Medical Microbiology, University of Zurich, Zurich, Switzerland; Institute of Medical Microbiology, University of Zurich, Zurich, Switzerland; Department of Microbiology, The University of Haripur, Haripur, Pakistan; Department of Microbiology, The University of Haripur, Haripur, Pakistan; Institute of Medical Microbiology, University of Zurich, Zurich, Switzerland; Institute of Medical Microbiology, University of Zurich, Zurich, Switzerland

## Abstract

**Background:**

Cefiderocol antimicrobial susceptibility testing requires standardized iron levels to produce consistent results. Here we assessed the analytical performances of two commercialized broth microdilution (BMD) methods (Bruker’s UMIC and Liofilchem’s ComASP), of disc diffusion (DD) and the gradient strip method on a collection of clinical carbapenem-resistant-*Pseudomonas aeruginosa* (CRPA) isolates.

**Methods:**

A collection of 117 WGS-CRPA was used. Cefiderocol susceptibility was determined (i) by manual BMD (reference method) using iron-depleted Mueller–Hinton (ID-MH), (ii) with the UMIC and ComASP BMD panels, (iii) by DD and (iv) using the gradient strip test, these latter two on bioMérieux MH-agar, Liofilchem MH-agar, and in-house-produced iron-depleted (ID)-MH agar plates.

**Results:**

Cefiderocol MIC_50_ and MIC_90_ were 1 and 4 mg/L, respectively. Eighteen isolates (15.4%) were classified as resistant (MIC > 2 mg/L) according to the standard EUCAST-based BMD. Overall, the ComASP panel showed the highest essential agreement (EA) with the standard BMD (82.1%), with a bias of −8.5%, and categorical agreement (CA) of 94%. The UMIC panel exhibited an EA of 74.4%, with a bias of 47%, and CA of 78.6%, exhibiting a tendency to overestimate the MICs. The EAs of the gradient strip test on bioMérieux-MH, Liofilchem-MH and ID-MH agar plates were 40.2%, 45.3% and 63.2%, respectively. Considering only categorizable results, the CAs of DD were 92.7%, 94.4% and 93.9%, respectively.

**Conclusions:**

The commercial ComASP panel may be used for cefiderocol testing of *P. aeruginosa* clinical isolates in diagnostic laboratories, ideally in combination with another method, such as the EUCAST-based DD. However, the standard manual BMD should still be performed to confirm the result when MICs are close to the breakpoint.

## Introduction

Therapeutic options for carbapenem-resistant *Pseudomonas aeruginosa* (CRPA) infections are often limited to polymyxins, aminoglycosides and fosfomycin.^1^ In recent years, several β-lactam/β-lactamase inhibitor combinations such as ceftolozane/tazobactam, ceftazidime/avibactam, imipenem/relebactam and meropenem/vaborbactam have been approved for clinical use.^2^ Although these compounds are effective against class A and class C β-lactamases, exhibiting good activity against non-carbapenemase-producing CRPA, they are ineffective against MBLs, such as the VIM enzymes, which are the most common carbapenemase type found in *P. aeruginosa* in Switzerland.^3^ Cefiderocol is a novel siderophore cephalosporin recently approved for treating a wide range of MDR Gram-negative pathogens, including CRPA.^4^ Worryingly, Cefiderocol-resistant CRPA are increasingly reported.^3^ Cefiderocol is structurally similar to ceftazidime and cefepime, and by means of its siderophore moiety it can bind to ferric ions and gain access into bacterial cells through active iron transporters.^5^ This unique feature, which allows it to overcome resistance mechanisms such as up-regulation of efflux pumps and porin loss, also poses a great challenge for antibiotic susceptibility testing (AST). In fact, accurate *in vitro* AST requires iron-depleted conditions (iron-depleted Mueller–Hinton; ID-MH) to induce siderophore-mediated entry. Whereas MIC determination with the standard broth microdilution (BMD) method requires the use of cation-adjusted ID-MH,^6^ disc diffusion (DD) does not require ID-MH medium, since the iron in the MH-agar is presumably bound to the agar, thereby mimicking the iron-limited environment.^7^ Further complicating cefiderocol AST, different clinical breakpoints (CBPs) for MIC and DD have been released from EUCAST and CLSI.^8,9^

The complexity in the preparation of ID-MH and the challenges in interpreting MICs due to trailing endpoints make the standard BMD method time consuming and not suitable for the daily workflow of a clinical microbiology laboratory. Cefiderocol Microdilution Panels such as ComASP^®^ (Liofilchem, Roseto degli Abruzzi, Italy) and UMIC^®^ (Bruker Daltonics, Germany) have recently been commercialized, but reports on their performance with CRPA are limited and show variable performances.^10–14^ Additionally, Cefiderocol MTS^TM^ (MIC Test strip, Liofilchem), specifically developed for *P. aeruginosa*, has recently been evaluated and is now commercially available. However, there are limited data on its performance with CRPA on MH agar plates, and a recent study discouraged its use with *P. aeruginosa*.^15^ Several works investigating the performance of DD with *P. aeruginosa* isolates have been published, reporting variable results depending on different media and disc producers.^15,16^ In this context, while maintaining the same CBP (susceptible  ≥22 mm; resistant <22 mm), in 2024 EUCAST significantly reduced the area of technical uncertainty (ATU) from 14–22 mm to 20–21 mm. This adjustment was based on a demonstrated strong correlation between BMD MICs and DD inhibition zones, validated across a broad collection of clinical isolates of *P. aeruginosa* (https://www.eucast.org/fileadmin/src/media/PDFs/EUCAST_files/Disk_criteria/Validation_2024/Pseudomonas_aeruginosa_v_10.0_January_2024.pdf). Also, in a recent study we showed that for *A. baumannii*, DD and gradient strip test on ID-MH agar plates produced more consistent results with the standard BMD method than on CAMHB agar plates.^17^

In this work, we aimed to compare the performance of gradient strip test and DD on MH from two different brands and an in-house-produced ID-MH plate, and of the commercially available BMD panels ComASP^®^ and UMIC^®^, using a collection of 117 CRPA clinical isolates.

## Materials and methods

### Strain collection

**Figure 1.**
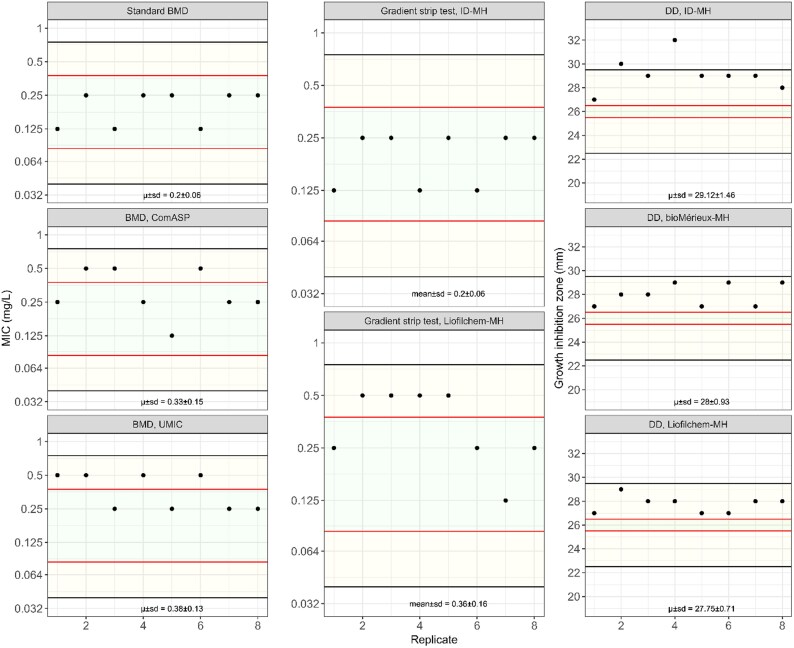
Quality control. Cefiderocol MIC values obtained with the standard BMD, ComASP, UMIC, and by gradient strip test on iron-depleted MH-agar (ID-MH), bioMérieux MH agar (bioMérieux-MH), Liofilchem MH agar (Liofilchem-MH) plates, and disc diffusion (DD) growth inhibition zones on ID-MH agar, bioMérieux-MH agar and Liofilchem-MH agar plates of *P. aeruginosa* ATCC27853. Replica numbers are on the *x*-axis and MICs on the *y*-axis. Green and yellow areas reflect the EUCAST targets and ranges for *P. aeruginosa* ATCC27853, respectively. μ, mean.

A collection of 117 non-duplicate CRPA isolates from various origins and exhibiting resistance to piperacillin/tazobactam, ceftazidime, cefepime, imipenem and/or meropenem was used in this study (Figure S1, available as Supplementary data at *JAC* Online). Ninety-five CRPA were isolated at the Institute of Medical Microbiology, University of Zurich (Zurich, Switzerland) as part of routine diagnostics, 10 at the Khyber Teaching Hospital, Peshawar (Pakistan), and 12 were obtained from the diverse panel of clinical *P. aeruginosa* isolates of the Walter Reed Army Institute of Research (Maryland, USA).^18^ The presence of β-lactamase genes was characterized by WGS (see below). *P. aeruginosa* ATCC27853 was included as a quality control strain in each experimental setting, to ensure that the quality control results of the AST were within the EUCAST ranges (Figure 1).

### Antimicrobial susceptibility testing

#### Disc diffusion

All 117 isolates were tested by the Kirby–Bauer DD method for susceptibility towards classic β-lactams (piperacillin/tazobactam, ceftazidime, cefepime, meropenem and imipenem), aminoglycosides (amikacin, gentamicin and tobramycin) and quinolones (ciprofloxacin and levofloxacin; see Figure S1). DD was performed on MH agar plates (bioMérieux, Marcy l’Étoile, France) according to the EUCAST guidelines.^19^ In addition, cefiderocol DD was performed on MH agar plates from a different manufacturer (Liofilchem, Roseti degli Abruzzi, Italy) and on home-made ID-MH agar plates, which were prepared using ID-MH produced according to the CLSI approved methodology:^20^ 50 g of Chelex^®^ 100 resin (Bio-Rad Laboratories, Hercules, CA, USA) were added to 1 L of autoclaved MHB (Merck KGaA, Darmstadt, Germany). The suspension was stirred for 2 h at room temperature (23°C) to remove cations in the medium. The iron-depleted broth was passed through a 0.2 μm filter to remove the resin. The pH of the broth was adjusted to 7.3 using 0.1 M hydrochloric acid. The ID-MH was supplemented with 22.5 μg/mL CaCl_2_ (range, 20–25 μg/mL), 11.25 μg/mL MgCl_2_ (range, 10–12.5 μg/mL) and 10 μM ZnSO_4_ (0.56 μg/mL; range 0.5–1.0 μg/mL). The solution was finally passed through a 0.2 μm filter for sterilization. For the preparation of ID-MH agar plates, 20 g of agar was added to 1 L MH. After sterilization, 25 mL of medium was dispensed into empty Petri dishes. Cefiderocol discs (30 µg) were purchased from Liofilchem. All other antibiotic discs were from Oxoid Limited (Basingstoke, UK). The inhibition zone diameters were measured with the SIRweb/SIRscan system (i2a).^21^

### MIC determination

#### Reference BMD

Reference MIC values were determined with the standard manual BMD method according to the EUCAST guidelines using ID-MH medium from Liofilchem (Roseti degli Abruzzi, Italy).^6^ The cefiderocol MICs were read after 18 h of incubation at 35°C.

#### Commercial BMD panels

The Bruker UMIC**^®^** and ComASP**^®^** Cefiderocol Microdilution Panels were performed according to the manufacturer’s instructions. Results were interpreted based on the EUCAST clinical breakpoint.^9^

#### Gradient strip test

Cefiderocol gradient strip test was performed using Cefiderocol MTS^™^ MIC Test Strip (cefiderocol 0.016–256 μg/mL; Liofilchem, Italy), MH agar plates from bioMérieux and Liofilchem, as well as home-made ID-MH agar plates, according to the manufacturer’s instructions (https://www.liofilchem.com/images/brochure/mic_test_strip_patent/MTS51.pdf). MIC values were rounded up and adjusted to a binary log scale (i.e. 0.002, (…), 128, 256). MICs for piperacillin/tazobactam, ceftazidime, ceftazidime/tazobactam, ceftolozane/tazobactam, cefepime and ceftriaxone were determined using MH agar plates. All gradient strips were obtained from Liofilchem. MICs were read after 18 h of incubation at 35°C.

### Data and bioinformatic analysis

Essential agreement (EA) was defined as MIC ± 1-fold dilution of the reference MIC (determined with the reference BMD method). Categorical agreement (CA) and clinical errors (major error, ME; very major error, vME) were determined on the basis of the EUCAST breakpoints.^9^ The expected performance criteria were as follows: for MIC-based methods, EA ≥ 90% and bias within ±30%, based on ISO 20776-2:2021;^22^ for disc diffusion, CA ≥ 90%, ME ≤ 3% and vME ≤ 1.5%, based on ISO 20776-2:2007.^23^ CA was defined as the percentage of isolates for which the test method and the reference BMD method yielded the same clinical category (‘S’, ‘I’ and ‘R’). MEs occurred when the test method reported resistance, whereas the reference method reported susceptibility. vMEs occurred when the test method reported susceptibility, whereas the reference method reported resistance. ME and vME rates were calculated using the number of reference-susceptible and reference-resistant isolates as denominators, respectively. Bias was calculated as the mean difference in MIC values (log₂ scale) between the test and reference methods.

#### WGS

WGS was performed at the IMM under ISO17025 accreditation. Bacterial genomic DNA was extracted using the DNeasy^®^ Ultraclean^®^ Microbial kit (Qiagen, Hilden, Germany) or Maxwell^®^ RSC Genomic DNA Kit using the blood DNA kit (Promega) according to the manufacturer’s instructions. Library preparation was conducted with the QIAGEN QIASeq FX kit (Qiagen, Hilden, Germany). Paired-end sequencing (2 × 150 bp) of DNA libraries was performed using an Illumina MiSeq or NextSeq1000 platform (Illumina^®^, San Diego, CA, USA) to a minimum mean read depth of 25×. MLST analysis was carried out using Ridom Seqsphere+. SNP analysis was performed in CLC Genomics Workbench v25.0, with variant calling performed using the parameters: 10 min coverage, 10 min count and 70% min frequency. SNP phylogenies were generated using neighbour joining and parameters: minimum coverage 10, no prune distance, minimum *z*-score 1.96, multi-nucleotide variants included.

### Detection of β-lactam resistance genes

Raw read data were processed through the IMMense pipeline (https://gitlab.uzh.ch/appliedmicrobiologyresearch/immense). This uses trimmomatic (version 0.39) to filter and trim raw sequencing data,^24^ and Unicycler v0.4.8 for assembly.^25^ Plasmid β-lactamase genes were identified querying NCBI-AMRfinderplus v3.12.8.^26^ All genome sequences generated at IMM, UZH (Table S1) were submitted to the European Nucleotide Archive (https://www.ebi.ac.uk/ena/browser) under project number PRJEB82838.

#### Cloning of bla_PAC-1_

To assess the impact of the AmpC-type β-lactamase PAC-1, the respective genes including their native promoter were amplified by PCR using the forward primer pre-PAC-1-for (5′- TATAGAATTCAACACGTCTCTACCCTGAATG-3′) and reverse primer pre-PAC-1-rev (5′-TATAGGATCCTCTCTATGCGGTTGGCTGTG-3′). The resulting PCR product of 1258 bp was cloned in the ZeroBlunt TOPO vector (Invitrogen, France) and then transformed in *Escherichia coli* XL-Blue electrocompetent cells (Agilent, USA).

## Results

### Genotypic analysis and cefiderocol standard AST

**Figure 2.**
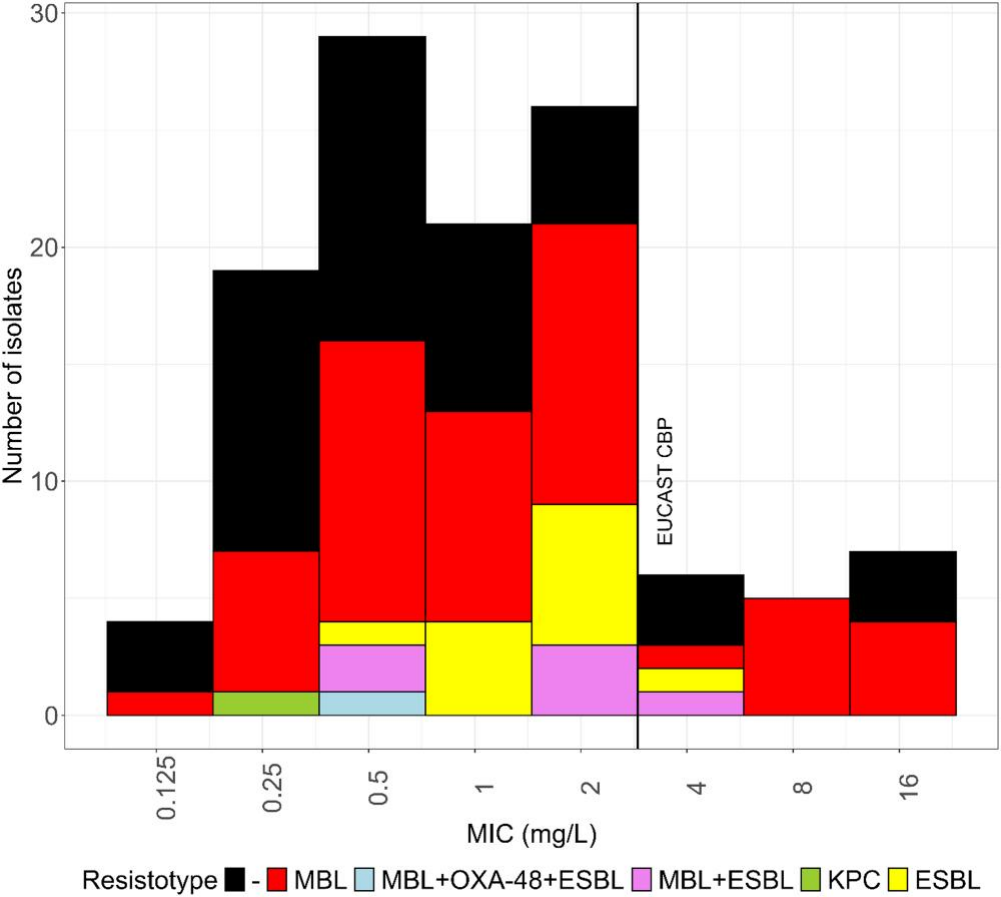
Distribution of cefiderocol MICs determined with the reference broth microdilution (BMD) method. The isolates were grouped according to the acquired carbapenem-resistance marker(s). The vertical line denotes the EUCAST clinical breakpoint. MIC readings were performed in accordance with the EUCAST guidance document on BMD testing of cefiderocol.

The strain collection included representative strains from 46 different STs, with ST235 (22/46, 47.8%) and ST111 (11/46, 23.9%) being the most prevalent (Table S1). Fifty-eight of 117 isolates (49.6%) harboured a gene coding for a carbapenemase, all but one belonging to the class B MBL group. The *bla*_VIM_ gene was the most common, present in 30/57 isolates (52.6%), followed by 16 *bla*_IMP_, 6 *bl*a_NDM_, 2 *bla*_IMP_/*bl*a_NDM_, 1 *bla*_VIM_/*bl*a_NDM_, 1 *bla*_IMP-26_/*bla*_OXA-181_, 1 *bla*_AIM-1_ and 1 *bla*_KPC-2_. In seven carbapenemase-producers, a gene encoding an ESBL was detected. A gene encoding an ESBL was also found in 12 carbapenemase-negative isolates: four *bla*_VEB_, five *bla*_GES_ and three *bla*_PER_. In the remaining 47 isolates (40.2%) no genes encoding carbapenem-resistance markers were detected. Cefiderocol MICs ranged from 0.125 to 16 mg/L (MIC_50_ = 1 mg/L, MIC_90_ = 4 mg/L), with 18 of 117 isolates (15.4%) exhibiting resistant MICs (>2 mg/L; see Figure 2 and Table S1). Among the isolates displaying high cefiderocol MICs (≥8 mg/L), eight belonged to the same ST (ST4936), were isolated at the Khyber Teaching Hospital in Peshawar, Pakistan, and were found to encode an IMP-1 carbapenemase and a plasmid-borne PAC-1 AmpC-type β-lactamase. SNP analysis revealed that these isolates clustered into three major groups containing five, one and two isolates, differing by approximately 250 and 900 SNPs, respectively (Figure S2).

### PAC-1 contribution to cefiderocol resistance

To investigate the underlying mechanism of cefiderocol resistance in isolates with high MICs, *bla*_PAC-1_ was cloned into a TOPO vector and expressed in *E. coli* (Table S2). Overexpression of PAC-1 led to increased MICs of ceftazidime, cefepime and ceftolozane/tazobactam. Susceptibility to piperacillin/tazobactam was only moderately reduced. Avibactam did not restore susceptibility to ceftazidime. Notably, PAC-1 overproduction resulted in a 5-fold increase in cefiderocol MIC, reaching 0.5 mg/L using standard BMD.

Genomic analysis of cefiderocol-resistant isolates revealed mutations in genes associated with iron uptake (e.g. *fecA* with *fecI/fecR*; *piuB* with *piuD*; *pirA* with *pirR/pirS*), as well as in *ftsI* (PBP3), the chromosomal *ampC* gene and its regulators (*ampD*, *ampR*). A loss-of-function mutation in *oprD* was also detected. These mutations were identical across all resistant isolates (Table S3).

### Gradient strip test

**Figure 3.**
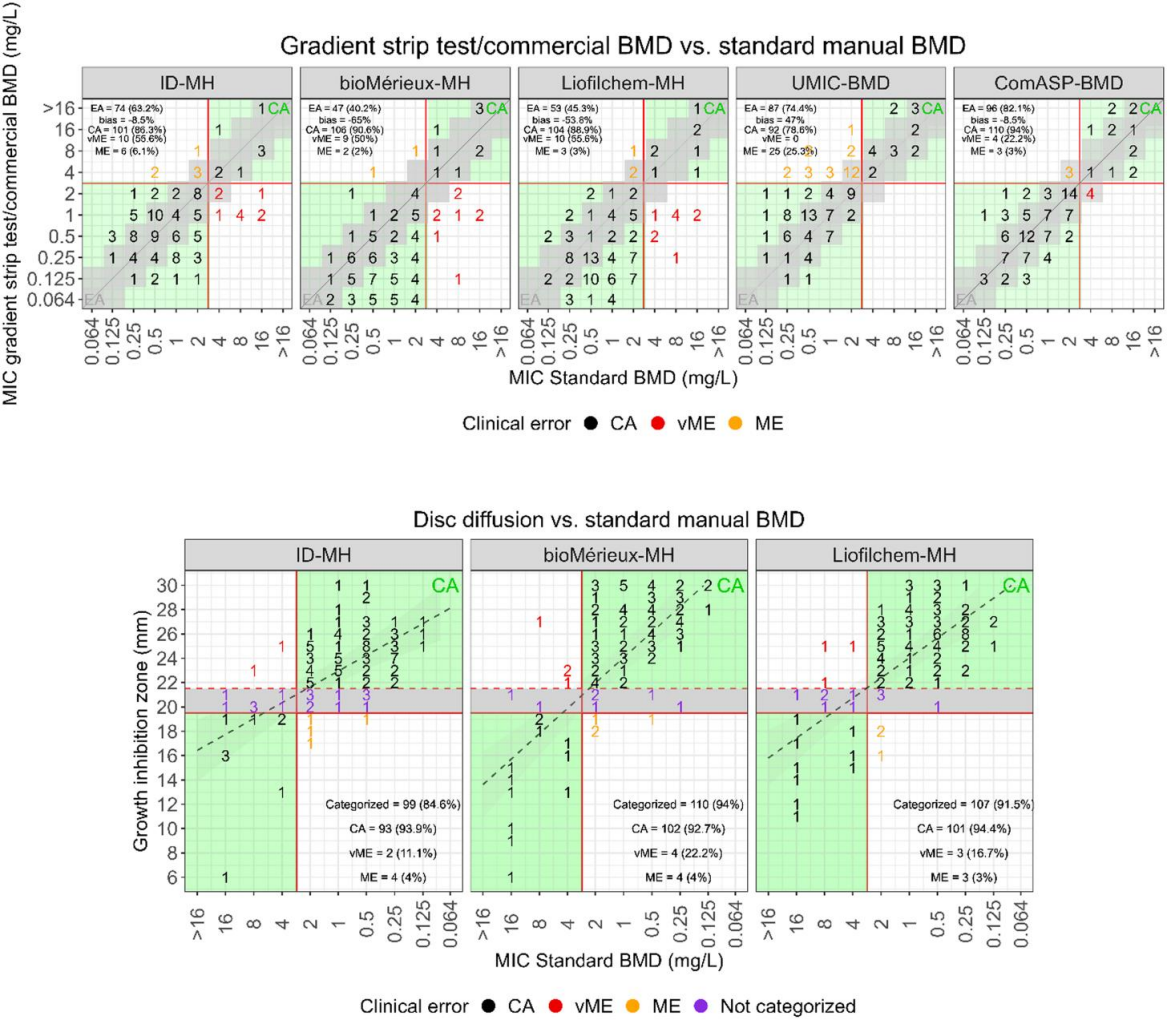
Gradient strip test, commercial BMD and disc diffusion versus standard BMD. MICs determined with the standard BMD method are on the *x*-axis. Gradient strip test/ComASP/UMIC MICs (top) and growth inhibition diameters (bottom) are on the *y*-axis. Isolates were categorized according to the BMD MICs and the EUCAST pharmacokinetic/pharmacodynamic (PK-PD) breakpoint. In the top figures, MICs/MICs are classified as categorical agreement (black), very major error (red) and major error (orange). The black diagonal dotted lines in the top figures denote identity between the methods. In the bottom figures, MICs/zone diameters are equally classified, with zone diameters within the ATU and thus not categorizable displayed in violet. The red continuous lines denote the EUCAST PK-PD breakpoint. The red dashed line in the bottom figures delimits the ATU. The black dashed lines in the bottom figures denote the regression lines. The green areas denote zones of congruence between the two methods, whereas the grey areas in the top figures indicate essential agreement. In the bottom figures, grey areas delimit the zone where inhibition diameters cannot be categorized (20–21 mm, according to the EUCAST guidelines).

Cefiderocol MICs of the *P. aeruginosa* ATCC27853 QC strain as determined by gradient strip test remained stable throughout the experimental settings and were always within the EUCAST range (0.064–0.5 mg/L, see Figure 1). The means of the MICs were 0.2 ± 0.06 mg/L, 0.22 ± 0.06 mg/L and 0.36 ± 0.16 mg/L when using the ID-MH, bioMérieux-MH and Liofilchem-MH agar plates, respectively. The highest EA with the standard BMD was obtained with the ID-MH agar plates (63.2%; 95% CI: 54.2%–72.4%) with a bias of −8.5%, whereas the EAs with the Liofilchem-MH and bioMérieux-MH agar plates were 45.3% (95% CI: 36.6%–54.3%) and 40.2% (95% CI: 31.7%–49.4%) with biases of −53.8% and −65%, respectively (Table 1, Figure 3). However, the rate of CA remained consistent when using the three different plates (between 86.3% and 90.6%; 95% CI: between 79.2%–91% and 83.7%–95%, respectively). Likewise, the rate of vMEs was very similar with the three types of MH agar plates (between 50% and 55.6%). Importantly, all the plates failed to detect a significant number of isolates exhibiting resistant MICs >2 mg/L, which were misclassified as susceptible.

**Table 1.** Performance of the AST methods

Method	Medium	EA	Bias	Categorized	CA	vME	ME
Gradient strip	ID-MH	74 (63.2%)	−8.50%	—	101 (86.3%)	10 (55.6%)	6 (6.1%)
	bioMérieux-MH	47 (40.2%)	−65%	—	106 (90.6%)	9 (50%)	2 (2%)
	Liofilchem-MH	53 (45.3%)	−53.80%	—	104 (88.9%)	10 (55.6%)	3 (3%)
UMIC-BMD	ID-MH	87 (74.4%)	47%	—	92 (78.6%)	0	25 (25.3%)
ComASP-BMD	ID-MH	96 (82.1%)	−8.50%	—	110 (94%)	4 (22.2%)	3 (3%)
Disc diffusion	ID-MH	—	—	99 (84.6%)	93 (93.9%)	2 (11.1%)	4 (4%)
	bioMérieux-MH	—	—	110 (94%)	102 (92.7%)	4 (22.2%)	4 (4%)
	Liofilchem-MH	—	—	107 (91.5%)	101 (94.4%)	3 (16.7%)	3 (3%)

BMD, broth microdilution; CA, categorical agreement; EA, essential agreement; ID-MH, iron-depleted Mueller–Hinton; ME, major error; MH, Mueller–Hinton; vME, very major error.

### Broth microdilution

Cefiderocol MICs for the *P. aeruginosa* ATCC27853 QC strain as determined with the UMIC and ComASP BMD panels fell always within the EUCAST range (0.064–0.5 mg/L, see Figure 1). The rates of EA and CA with the standard BMD of the UMIC panel were 74.4% (95% CI: 66.5%–82.3%) and 78.6% (95% CI: 66.5%–82.3%), respectively (Table 1, Figure 3). The UMIC panel exhibited MICs on average higher than the standard BMD method. This resulted in a high bias with the standard BMD method and a high rate of MEs (25.3%). Notably, all 18 resistant isolates were correctly categorized (no vMEs). The EA and CA of the ComASP panel were 82.1% (95% CI: 71.4%–85.1%) with a bias of −8.5% (95% CI: −4.4% to −15.6%), and 94% (95% CI: 89.5%–97.5%), respectively. The ComASP panel caused four vMEs (22.2%) and three MEs (3%), the majority of which occurred with isolates with MICs close to the breakpoint.

### Disc diffusion

Cefiderocol inhibition zones of the *P. aeruginosa* ATCC27853 QC strain remained stable throughout the experimental settings, with differences depending on the medium (Figure 1). Although the inhibition zones were always within the EUCAST cefiderocol QC range (range 23–29 mm, target 26 mm) when tested on bioMérieux (28 ± 0.9 mm) and Liofilchem (27.8 ± 0.7 mm) MH agar plates, when using the homemade ID-MH agar plate the values fell in two cases outside the acceptable range (29.1 ± 1.5 mm). Overall, the rate of categorizable values with the standard BMD method ranged between 84.6% when using the homemade ID-MH agar plates to 91.5%–94% when using the commercial MH plates (Figure 3). When considering the categorized values, the rate of CA was 92.7% (95% CI: 87%–96.2%) with the bioMérieux-MH plates, 94.4% (95% CI: 89%–97.3%) with the Liofilchem-MH plates, and 93.9% (95% CI: 88.7%–96.9%) with the ID-MH plates. The rate of vMEs varied from 11.1% with the ID-MH plates, to 16.3% with Liofilchem-MH plates, and 22.2% with the bioMérieux-MH plates. The rate of MEs was 3% with the Liofilchem-MH plates, and 4% with both the bioMérieux-MH and ID-MH agar plates. Of note, all the MH agar plates under investigation failed to detect some resistant strains exhibiting MICs >4 mg/L. Finally, 2/18 isolates misclassified as susceptible when using the ID-MH plates, 4/18 when using the bioMérieux-MH plates and 3/18 when using the Liofilchem-MH plates.

## Discussion

This study evaluated the performance of DD, gradient strips and two commercial BMD panels in assessing the susceptibility of 117 *P. aeruginosa* clinical isolates multi-resistant to cefiderocol. The ComASP panel exhibited higher analytical performance (EA 82.1% and CA 94%) compared with the UMIC panel (EA 74.4% and CA 78.6%). However, their performances did not meet the criteria outlined in ISO 20776-2:2021 (EA and CA ≥90%, bias ±30%).^22^ The UMIC panel tended to overestimate the MICs, resulting in a high rate of MEs (25.3%), but no vMEs. In contrast, MICs obtained with the ComASP panel showed a better correlation with the standard method and resulted in similar rates of ME and vME, primarily due to values close to the breakpoint. Based on these findings, our recommendations are that the ComASP panel may be used for cefiderocol testing of *P. aeruginosa* clinical isolates in diagnostic laboratories. However, if the MICs fall within a ±1-fold difference from the CBP, the standard BMD should still be performed for confirmation.

Gradient strips achieved CA rates (86.3%–90.6%) and EA rates (40.2%–63.2%) below acceptability (≥90%). Importantly, none of them allowed the reliable detection of resistant isolates exhibiting MICs ≥4 mg/L. Disappointingly, the ID-MH agar plates did not improve the detection of resistance isolates, as previously reported for *A. baumannii*, where these plates favoured the emergence of resistant subpopulations within the inhibition zones, thereby increasing the MICs and improving the congruence with the standard method.^17^ Based on these findings, and in line with previous reports, gradient strips cannot be recommended for cefiderocol testing in *P. aeruginosa*.^15^

In 2024 EUCAST reduced the ATU from 14–22 mm to 20–21 mm, while maintaining the CBP of 22 mm. According to these new guidelines and considering only the inhibition zones falling outside the ATU, DD achieved rates of CA with the standard method within the acceptable value (92.7%–94.4%). Importantly, despite some resistant isolates being either misclassified as susceptible or falling within the ATU zone, the method appeared to be more robust than the gradient strip and may therefore be considered for use in clinical laboratories, particularly to confirm high-level resistance.

The strengths of this study were the genetic diversity of the collection, which included 46 different STs, and the relatively high number of cefiderocol-resistant isolates (18/117, 15.4%), allowing a robust evaluation of the different methods in detecting resistance. However, the distribution of cefiderocol MICs among the tested isolates also represents a limitation. Most strains were cefiderocol-susceptible, with a MIC_90_ of 4 mg/L, which is relatively low. This skewed distribution likely amplified the impact of MIC overestimation by the UMIC panel on both categorical and essential agreement. Interestingly, ESBL producers were not associated with high cefiderocol MICs, an association that has previously been reported for *A. baumannii*.^17^

Among strains with cefiderocol MICs >4 mg/L, 8 of 16 produced a class C β-lactamase (AmpC-type), which has been previously shown to confer resistance in *P. aeruginosa* to ceftazidime, cefepime, ceftolozane/tazobactam and ceftazidime/tazobactam, while only partially affecting piperacillin/tazobactam susceptibility.^27^ Heterologous overproduction of PAC-1 in *E. coli* confirmed these findings and suggested that PAC-1 overexpression may contribute to reduced cefiderocol susceptibility. However, since the resulting cefiderocol MIC (0.5 mg/L) remains within the susceptible range, PAC-1 alone is unlikely to confer full resistance. Additional mutations detected in genes previously associated with reduced cefiderocol susceptibility^28–30^ may act synergistically with PAC-1 to increase MICs. Their precise impact remains unclear and warrants further functional validation.

Due to its specific chemical features and the complexity arising from the challenges of standardizing the peculiar AST conditions, more studies are warranted to assess the effectiveness of the available AST methods. Additionally, exploring different media compositions may help to identify optimal test conditions. These studies should involve the use of large and diverse collections of strains, covering the entire spectrum of susceptibility to cefiderocol.

## Supplementary Material

dkaf301_Supplementary_Data

## Supplementary data

Figures S1 and S2 and Tables S1 to S3 are available as Supplementary data at *JAC* Online.
