# Supplementary material for: Performance of disc diffusion and gradient strip test on different Mueller–Hinton agar media plates and ComASP® and Bruker UMIC® Cefiderocol Microdilution Panels for cefiderocol susceptibility testing in carbapenem-resistant Pseudomonas aeruginosa
Source: J Antimicrob Chemother. 2025 Aug 18;80(10):2799–806. doi: 10.1093/jac/dkaf301 (PMC12494204; doi:10.1093/jac/dkaf301)
Supplement: dkaf301_Supplementary_Data [file dkaf301_supplementary_data.docx]

**Supplementary data**

**Figures**


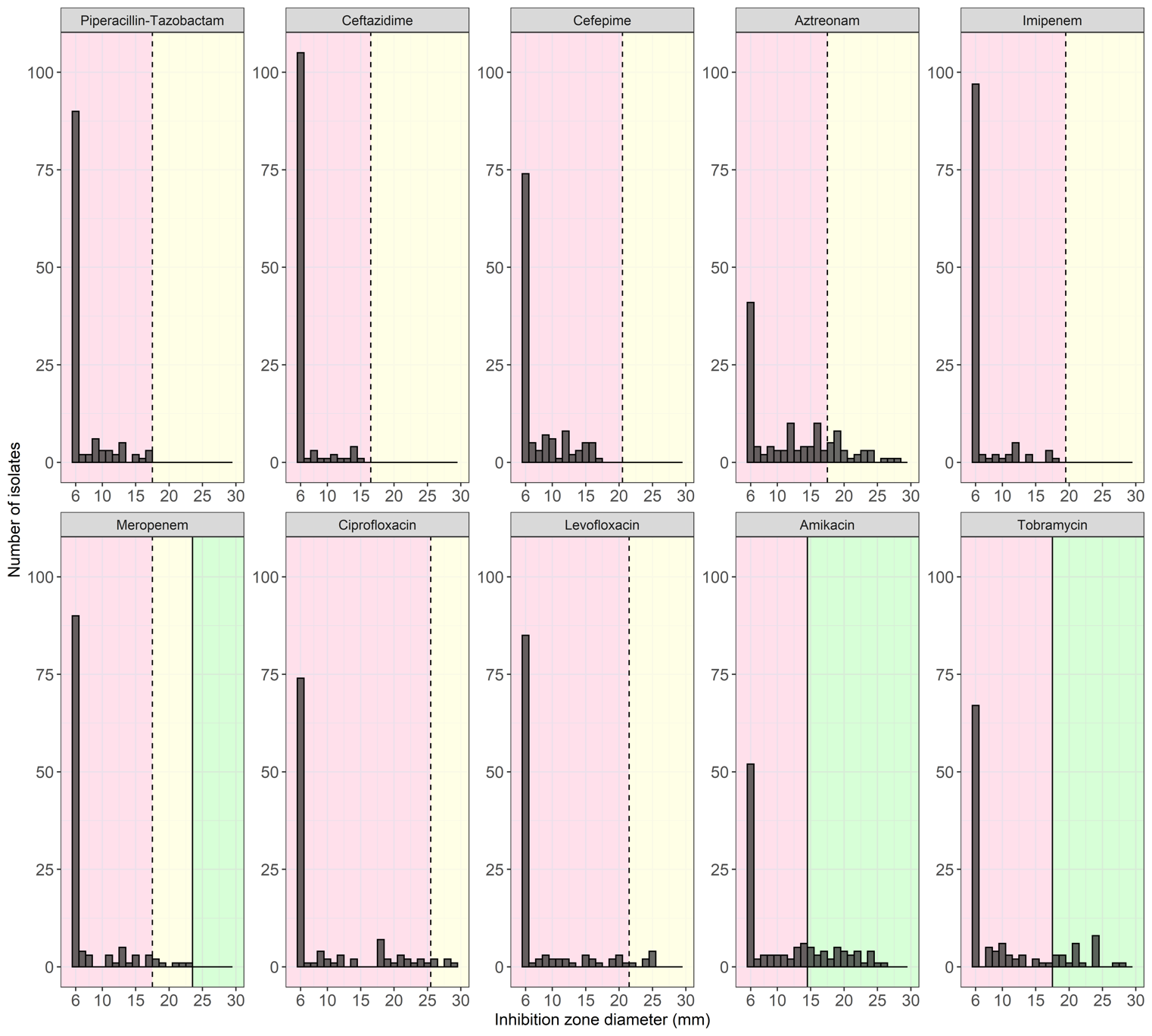
**Figure S1 Distribution of growth inhibition zones of first-line antibiotics.** The vertical continuous and dashed lines denote the EUCAST CBPs. The red, yellow, and green zones indicate resistance, the ‘susceptibility by increased exposure’ category, and susceptibility.

**Figure S2. Phylogeny of ST4936 isolates from Pakistan. A.** Neighbor joining tree based on SNPs called using CLC Genomics Workbench (see methods) using the Unicycler assembly of CRPAZU010 as a reference. **B.** Distance matrix showing SNPs between isolates in pairs, illustrating scale in **A.**

**A.**

**
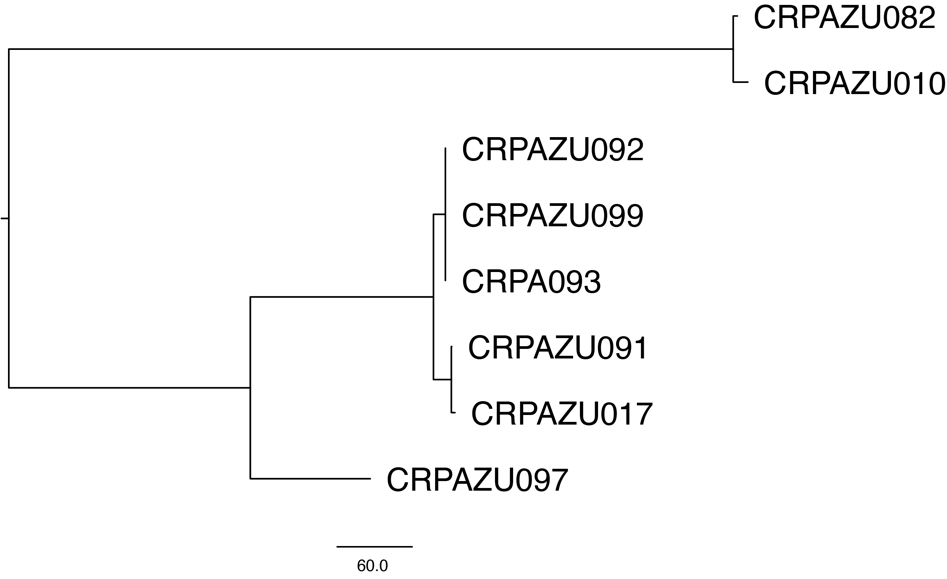
**

**B.**

**Tables**

**Table S1. Sequence type (ST), beta-lactam resistance markers and cefiderocol AST data of the isolates included in the study.**

| **CRPAZU N.** | **ST** | **Carbapenemase(s)** | **ESBL** | **PDC** | **Intrinsic OXA** | **Other plasmid-based β-lactamases** | **Origin** | **Reference BMD MIC (mg/L)** | **Commercial BMD panels MIC (mg/L)** | | **Gradient strip test MIC (mg/L)** | | | **DD growth inhibition zones (mm)** | | |
| --- | --- | --- | --- | --- | --- | --- | --- | --- | --- | --- | --- | --- | --- | --- | --- | --- |
|  |  |  |  |  |  |  |  |  | **ComASP®** | **Bruker UMIC®** | **bioMérieux MH** | **Liofilchem MH** | **ID-MH-agar** | **bioMérieux MH** | **Liofilchem MH** | **ID-MH-agar** |
| CRPAZU002 | 664 | - | GES-12 | PDC-98 | OXA-50 | - | IMM, UZH | 1 | 0.5 | 0.5 | 0.032 | 0.094 | 0.38 | 30 | 28 | 24 |
| CRPAZU003 | 2572 | - | - | PDC-374 | OXA-488 | - | IMM, UZH | 0.5 | 0.5 | 0.5 | 0.19 | 0.25 | 0.75 | 26 | 27 | 25 |
| CRPAZU004 | 235 | VIM-4 | - | PDC-35 | OXA-488 | OXA-35/CARB-2 | IMM, UZH | 0.25 | 0.25 | 0.125 | 0.125 | 0.25 | 0.75 | 26 | 28 | 24 |
| CRPAZU005 | 941 | - | - | PDC-374 | OXA-396 | - | IMM, UZH | 0.25 | 0.5 | 1 | 2 | 1 | 2 | 20 | 23 | 24 |
| CRPAZU006 | 485 | - | - | PDC-6 | OXA-50 | - | IMM, UZH | 2 | 2 | 4 | 1.5 | 3 | 3 | 21 | 22 | 21 |
| CRPAZU007 | 245 | IMP-18 | - | PDC-5 | OXA-494 | OXA-10 | IMM, UZH | 0.5 | 0.5 | 1 | 0.125 | 0.25 | 0.25 | 29 | 26 | 25 |
| CRPAZU008 | 282 | VIM-4 | - | PDC-103 | OXA-50 | CARB-2 | IMM, UZH | 0.5 | 1 | 1 | 0.25 | 0.125 | 0.5 | 28 | 27 | 25 |
| CRPAZU009 | 357 | IMP-7 | - | PDC-11 | OXA-846 | OXA-2 | IMM, UZH | 2 | 2 | 2 | 6 | 6 | 1 | 18 | 16 | 22 |
| CRPAZU010 | 4936 | IMP-1 | - | PDC-35 | OXA-50 | OXA-10/PAC-1 | Haripur, Pakistan | **16** | 32 | 8 | 1 | 0.75 | 0.75 | 21 | 21 | 21 |
| CRPAZU011 | 285 | - | - | PDC-1 | OXA-494 | - | IMM, UZH | 0.25 | 2 | 2 | 0.38 | 0.38 | 0.38 | 25 | 25 | 23 |
| CRPAZU012 | 385 | - | - | PDC-5 | OXA-396 | - | IMM, UZH | 1 | 1 | 0.5 | 0.023 | 0.047 | 0.25 | 31 | 30 | 26 |
| CRPAZU013 | 111 | VIM-2 | - | PDC-3 | OXA-395 | - | IMM, UZH | 2 | 2 | 2 | 0.094 | 0.125 | 0.75 | 29 | 27 | 25 |
| CRPAZU014 | 316 | - | - | PDC-36 | OXA-395 | OXA-10 | IMM, UZH | 0.5 | 1 | 0.5 | 0.125 | 0.25 | 0.38 | 28 | 25 | 25 |
| CRPAZU015 | 316 | - | - | PDC-36 | OXA-395 | OXA-10 | IMM, UZH | 0.25 | 1 | 0.5 | 0.094 | 0.25 | 0.75 | 27 | 26 | 25 |
| CRPAZU016 | 3416 | - | - | PDC-23 | OXA-848 | - | IMM, UZH | 0.5 | 1 | 0.25 | 0.125 | 0.19 | 0.25 | 28 | 29 | 27 |
| CRPAZU017 | 4936 | IMP-1 | - | PDC-35 | OXA-50 | OXA-10/PAC-1 | Haripur, Pakistan | **16** | 256 | 64 | 24 | 8 | 512 | 9 | 14 | 6 |
| CRPAZU018 | 316 | - | - | PDC-36 | OXA-395 | OXA-10 | IMM, UZH | 0.25 | 1 | 0.5 | 0.125 | 0.19 | 0.5 | 29 | 28 | 26 |
| CRPAZU019 | 357 | OXA-181/IMP-26 | VEB-9 | PDC-3 | OXA-395 | OXA-21 | IMM, UZH | 0.5 | 2 | 8 | 3 | 1.5 | 2 | 19 | 20 | 19 |
| CRPAZU020 | 3416 | - | - | PDC-23 | OXA-848 | - | IMM, UZH | 0.5 | 1 | 0.25 | 0.064 | 0.125 | 0.38 | 29 | 26 | 25 |
| CRPAZU021 | 111 | VIM-2 | - | PDC-3 | OXA-395 | - | IMM, UZH | 2 | 2 | 2 | 0.094 | 0.125 | 0.25 | 28 | 31 | 26 |
| CRPAZU022 | 2572 | - | - | PDC-3 | OXA-488 | - | IMM, UZH | 0.25 | 0.25 | 0.5 | 0.19 | 0.19 | 0.38 | 29 | 27 | 25 |
| CRPAZU023 | 941 | - | - | PDC-374 | OXA-396 | - | IMM, UZH | 1 | 1 | 2 | 0.25 | 0.38 | 0.75 | 24 | 23 | 24 |
| CRPAZU024 | 664 | - | GES-12 | PDC-98 | OXA-50 | - | IMM, UZH | 1 | 0.5 | 0.5 | 0.032 | 0.047 | 0.75 | 28 | 28 | 24 |
| CRPAZU025 | 111 | VIM-4 | - | PDC-3 | OXA-395 | OXA-9/CARB-2 | IMM, UZH | 0.5 | 0.5 | 1 | 0.75 | 0.19 | 0.75 | 26 | 24 | 23 |
| - | 385 | - | - | PDC-374 | OXA-396 | - | IMM, UZH | 1 | 1 | 1 | 0.064 | 0.064 | 0.25 | 28 | 28 | 24 |
| CRPAZU026 | 189 | - | - | PDC-374 | OXA-905 | - | IMM, UZH | 1 | 2 | 1 | 1 | 0.75 | 1 | 25 | 26 | 23 |
| CRPAZU027 | 485 | - | - | PDC-374 | OXA-50 | - | IMM, UZH | 0.5 | 0.5 | 1 | 0.38 | 0.75 | 3 | 28 | 30 | 24 |
| CRPAZU028 | 235 | VIM-2 | - | PDC-35 | OXA-488 | - | IMM, UZH | 0.25 | 0.125 | 0.5 | 0.047 | 0.094 | 0.5 | 28 | 27 | 24 |
| CRPAZU029 | 111 | VIM-2 | - | PDC-3 | OXA-395 | - | IMM, UZH | 2 | 1 | 1 | 0.064 | 0.125 | 0.25 | 30 | 28 | 25 |
| CRPAZU030 | 1047 | IMP-1 | - | PDC-12 | OXA-488 | OXA-10 | IMM, UZH | 2 | 4 | 2 | 0.19 | 0.25 | 0.75 | 25 | 24 | 22 |
| CRPAZU031 | 111 | - | - | PDC-3 | OXA-395 | OXA-14/CARB-2 | IMM, UZH | 2 | 2 | 2 | 0.19 | 0.25 | 0.5 | 25 | 25 | 23 |
| CRPAZU033 | 988 | - | - | PDC-23 | OXA-904 | - | IMM, UZH | 0.25 | 0.25 | 0.5 | 0.023 | 0.064 | 0.094 | 31 | 31 | 31 |
| CRPAZU034 | 274 | - | - | PDC-24 | OXA-486 | - | IMM, UZH | 0.25 | 0.5 | 0.5 | 0.19 | 0.38 | 0.5 | 26 | 23 | 24 |
| CRPAZU035 | 253 | - | - | PDC-34 | OXA-488 | - | IMM, UZH | 0.25 | 0.125 | 0.25 | 0.008 | 0.064 | 0.25 | 34 | 32 | 27 |
| CRPAZU036 | 111 | VIM-2 | - | PDC-3 | OXA-395 | - | IMM, UZH | 1 | 2 | 0.5 | 0.047 | 0.094 | 0.38 | 30 | 30 | 26 |
| CRPAZU037 | 4072 | - | - | PDC-374 | OXA-486 | - | IMM, UZH | **4** | 4 | 8 | 6 | 6 | 3 | 16 | 16 | 19 |
| CRPAZU038 | 235 | VIM-4 | - | PDC-35 | OXA-488 | OXA-35/CARB-2 | IMM, UZH | 0.5 | 0.5 | 1 | 0.125 | 0.25 | 0.75 | 27 | 25 | 22 |
| CRPAZU039 | 1420 | - | - | PDC-19a | OXA-395 | - | IMM, UZH | 0.125 | 0.125 | 0.25 | 0.047 | 0.094 | 0.38 | 30 | 27 | 27 |
| CRPAZU040 | 485 | - | - | PDC-6 | OXA-50 | - | IMM, UZH | **4** | 2 | 4 | 3 | 4 | 1.5 | 17 | 18 | 21 |
| CRPAZU041 | 235 | VIM-4 | - | PDC-35 | OXA-488 | CARB-2 | IMM, UZH | 0.25 | 0.25 | 1 | 0.19 | 0.25 | 0.75 | 30 | 26 | 22 |
| CRPAZU042 | 27 | - | - | PDC-15 | OXA-494 | - | IMM, UZH | 2 | 2 | 16 | 1 | 1.5 | 6 | 22 | 21 | 20 |
| CRPAZU044 | n.t. | - | - | PDC-432 | OXA-1124 | - | IMM, UZH | 0.5 | 0.125 | 0.125 | 0.094 | 0.125 | 0.75 | 33 | 30 | 29 |
| CRPAZU045 | 235 | - | VEB-16 | PDC-35 | OXA-488 | OXA-10 | IMM, UZH | **4** | 16 | 8 | 0.38 | 0.75 | 1.5 | 23 | 20 | 20 |
| CRPAZU046 | 235 | VIM-4 | - | PDC-35 | OXA-488 | OXA-35/CARB-2 | IMM, UZH | 0.25 | 0.5 | 1 | 0.19 | 0.38 | 0.75 | 27 | 26 | 22 |
| CRPAZU047 | 111 | - | - | PDC-3 | OXA-395 | OXA-9 | IMM, UZH | 0.5 | 0.5 | 0.5 | 0.064 | 0.125 | 0.75 | 30 | 30 | 26 |
| - | 111 | - | - | PDC-55 | OXA-395 | OXA-9 | IMM, UZH | 0.125 | 0.125 | 1 | 0.064 | 0.125 | 0.5 | 30 | 27 | 26 |
| CRPAZU048 | 357 | - | VEB-9 | PDC-11 | OXA-846 | OXA-10 | IMM, UZH | 2 | 1 | 2 | 0.75 | 0.75 | 1.5 | 24 | 23 | 23 |
| CRPAZU049 | 235 | AIM-1 | SHV-5 | PDC-35 | OXA-488 | - | IMM, UZH | 0.5 | 0.25 | 0.5 | 0.38 | 0.25 | 0.38 | 30 | 23 | 23 |
| - | n.t. | - | - | PDC-167 | OXA-902 | - | IMM, UZH | 0.5 | 0.125 | 0.25 | 0.008 | 0.047 | 0.25 | 35 | 35 | 31 |
| CRPAZU050 | 235 | VIM-28 | SHV-2a | PDC-35 | OXA-488 | - | IMM, UZH | 0.5 | 0.5 | 4 | 0.38 | 1.5 | 1 | 21 | 22 | 21 |
| CRPAZU051 | 111 | VIM-4 | - | PDC-3 | OXA-395 | OXA-9/CARBA-2 | IMM, UZH | 0.5 | 0.5 | 2 | 0.064 | 0.25 | 0.75 | 30 | 28 | 25 |
| CRPAZU052 | 111 | - | - | PDC-3 | OXA-395 | OXA-9 | IMM, UZH | 0.25 | 0.25 | 4 | 0.064 | 0.125 | 0.19 | 30 | 26 | 26 |
| CRPAZU053 | 235 | VIM-2 | - | PDC-35 | OXA-488 | - | IMM, UZH | 0.5 | 0.25 | 1 | 0.25 | 0.25 | 1 | 27 | 25 | 25 |
| CRPAZU054 | 235 | VIM-4 | - | PDC-35 | OXA-488 | OXA-35/CARB-2 | IMM, UZH | 1 | 0.5 | 2 | 0.19 | 0.38 | 1 | 27 | 25 | 23 |
| CRPAZU055 | 235 | VIM-4 | - | PDC-35 | OXA-488 | OXA-35/CARB-2 | IMM, UZH | 0.25 | 0.5 | 1 | 0.125 | 0.25 | 0.25 | 28 | 26 | 26 |
| - | 1171 | - | - | PDC-374 | OXA-50 | - | IMM, UZH | 0.125 | 1 | 2 | 0.25 | 0.38 | 0.25 | 31 | 33 | 33 |
| CRPAZU056 | 175 | VIM-1 | - | PDC-1 | OXA-50 | - | IMM, UZH | 2 | 0.5 | 8 | 2 | 4 | 1.5 | 18 | 18 | 23 |
| CRPAZU057 | 235 | - | - | PDC-35 | OXA-488 | OXA-35/CARB-2 | IMM, UZH | 0.5 | 0.25 | 2 | 0.047 | 0.125 | 0.38 | 30 | 28 | 27 |
| CRPAZU058 | 164 | - | - | PDC-121 | OXA-486 | - | IMM, UZH | 1 | 0.25 | 0.5 | 0.023 | 0.094 | 0.19 | 30 | 27 | 26 |
| CRPAZU059 | 357 | - | VEB-9 | PDC-11 | OXA-846 | OXA-10 | IMM, UZH | 2 | 2 | 4 | 0.38 | 0.75 | 0.75 | 23 | 24 | 23 |
| CRPAZU060 | 235 | VIM-4 | - | PDC-35 | OXA-488 | OXA-35/CARB-2 | IMM, UZH | 0.25 | 1 | 1 | 0.19 | 0.25 | 0.5 | 27 | 26 | 23 |
| CRPAZU061 | 235 | - | GES-52 | PDC-35 | OXA-488 | - | IMM, UZH | 2 | 1 | 4 | 0.064 | 0.19 | 0.38 | 30 | 25 | 24 |
| CRPAZU062 | 635 | - | - | PDC-8 | OXA-395 | - | IMM, UZH | 2 | 4 | 2 | 0.047 | 0.094 | 0.125 | 30 | 27 | 25 |
| CRPAZU063 | 235 | VIM-2 | - | PDC-35 | OXA-488 | OXA-10 | IMM, UZH | 0.5 | 0.5 | 1 | 0.19 | 0.19 | 0.38 | 25 | 26 | 21 |
| CRPAZU064 | 316 | - | - | PDC-36 | OXA-395 | OXA-10 | IMM, UZH | 0.25 | 0.5 | 1 | 0.25 | 0.25 | 0.38 | 26 | 25 | 25 |
| CRPAZU065 | 316 | - | - | PDC-36 | OXA-395 | OXA-10 | IMM, UZH | 0.5 | 0.5 | 1 | 0.064 | 0.19 | 0.38 | 26 | 26 | 25 |
| CRPAZU066 | 111 | VIM-2 | - | PDC-3 | OXA-395 | - | IMM, UZH | 2 | 4 | 4 | 0.047 | 0.125 | 0.5 | 27 | 27 | 25 |
| CRPAZU067 | 235 | NDM-1/VIM-2 | - | PDC-35 | OXA-488 | - | IMM, UZH | 0.5 | 0.125 | 1 | 0.19 | 0.25 | 1.5 | 26 | 24 | 21 |
| CRPAZU068 | 2553 | IMP-1 | - | PDC-3 | OXA-486 | - | IMM, UZH | 0.125 | 0.125 | 0.5 | 0.125 | 0.38 | 0.5 | 28 | 25 | 25 |
| CRPAZU069 | 155 | - | - | PDC-5 | OXA-396 | - | IMM, UZH | 0.25 | 0.25 | 4 | 0.064 | 0.064 | 0.38 | 29 | 26 | 24 |
| CRPAZU070 | 266 | - | - | PDC-1 | OXA-486 | - | IMM, UZH | 0.5 | 0.25 | 1 | 0.032 | 0.125 | 0.125 | 31 | 28 | 29 |
| CRPAZU071 | 357 | VIM-5 | VEB-14 | PDC-11 | OXA-846 | OXA-10 | IMM, UZH | 2 | 2 | 4 | 1 | 1 | 2 | 21 | 21 | 21 |
| CRPAZU072 | 1047 | IMP-1 | - | PDC-12 | OXA-488 | OXA-10 | IMM, UZH | 1 | 1 | 2 | 0.125 | 0.25 | 0.5 | 25 | 25 | 23 |
| CRPAZU073 | 235 | VIM-4 | - | PDC-35 | OXA-488 | CARB-2 | IMM, UZH | 1 | 0.5 | 1 | 0.094 | 0.125 | 0.5 | 28 | 28 | 23 |
| CRPAZU074 | 654 | VIM-2 | - | PDC-3 | OXA-396 | - | IMM, UZH | 0.5 | 0.25 | 1 | 0.094 | 0.38 | 0.5 | 29 | 29 | 27 |
| CRPAZU075 | 179 | - | PER-1 | PDC-8 | OXA-396 | OXA-4 | IMM, UZH | 0.5 | 2 | 4 | 0.38 | 0.125 | 3 | 25 | 25 | 19.5 |
| CRPAZU076 | 773 | NDM-1 | - | PDC-16 | OXA-395 | - | IMM, UZH | **4** | 2 | 4 | 1 | 0.5 | 3 | 22 | 21 | 19 |
| CRPAZU077 | 233 | VIM-5 | - | PDC-3 | OXA-486 | OXA-4 | IMM, UZH | 1 | 1 | 4 | 0.38 | 2 | 2 | 24 | 25 | 21 |
| CRPAZU078 | 773 | NDM-1 | - | PDC-16 | OXA-395 | - | IMM, UZH | 2 | 2 | 4 | 1 | 0.75 | 2 | 20 | 22 | 19.5 |
| - | 1125 | - | - | PDC-35 | OXA-395 | - | IMM, UZH | 1 | 0.5 | 0.5 | 0.047 | 1 | 0.25 | 28 | 27 | 25 |
| CRPAZU079 | 316 | VIM-2 | - | PDC-36 | OXA-395 | OXA-10/OXA-796 | IMM, UZH | 0.5 | 0.5 | 1 | 0.016 | 0.125 | 0.25 | 31 | 27 | 26 |
| CRPAZU080 | 654 | VIM-2 | - | PDC-3 | OXA-396 | - | IMM, UZH | 1 | 0.25 | 1 | 0.064 | 0.64 | 0.19 | 30 | 30 | 28 |
| CRPAZU081 | 1047 | IMP-1 | - | PDC-12 | OXA-488 | OXA-10 | IMM, UZH | **8** | 4 | 8 | 0.125 | 0.25 | 1 | 27 | 25 | 23 |
| CRPAZU082 | 4936 | IMP-1 | - | PDC-35 | OXA-50 | OXA-10/PAC-1 | Haripur, Pakistan | **16** | 16 | 32 | 0.75 | 0.75 | 0.75 | 14 | 19 | 20 |
| CRPAZU083 | 1047 | IMP-1 |  | PDC-12 | OXA-488 | OXA-10 | IMM, UZH | 0.5 | 0.5 | 4 | 0.38 | 0.25 | 1 | 24 | 23 | 24 |
| CRPAZU084 | 235 | NDM-1 | PME-1 | PDC-35 | OXA-488 | - | IMM, UZH | 2 | 1 | 4 | 0.5 | 0.5 | 0.5 | 25 | 25 | 24 |
| CRPAZU085 | 2167 | - | - | PDC-309 | OXA-1125 | - | IMM, UZH | 4 | 2 | 8 | 1 | 0.5 | 1 | 23 | 25 | 25 |
| - | 255 | - | - | PDC-55 | OXA-905 | - | IMM, UZH | **16** | 8 | 8 | 8 | 12 | 6 | 13 | 17 | 16 |
| CRPAZU086 | 244 | NDM-1 | PME-1 | PDC-1 | OXA-847 | OXA-10 | IMM, UZH | 2 | 1 | 4 | 1.5 | 1.5 | 3 | 22 | 18 | 22 |
| CRPAZU087 | 244 | NDM-1 | PME-1 | PDC-374 | OXA-847 | OXA-10 | IMM, UZH | **4** | 2 | 8 | 12 | 8 | 12 | 13 | 15 | 13 |
| - | 255 | - | - | PDC-55 | OXA-905 | - | IMM, UZH | **16** | 4 | 16 | 32 | 24 | 8 | 10 | 11 | 16 |
| - | 255 | - | - | PDC-55 | OXA-905 | - | IMM, UZH | **16** | 8 | 16 | 48 | 16 | 8 | 6 | 12 | 16 |
| CRPAZU088 | 235 | IMP-1 | - | PDC-35 | OXA-488 | - | IMM, UZH | 2 | 0.5 | 4 | 0.094 | 0.125 | 0.38 | 28 | 26 | 25 |
| CRPAZU089 | 664 | VIM-4 | - | PDC-98 | OXA-50 | - | IMM, UZH | 1 | 0.5 | 1 | 0.125 | 0.25 | 0.19 | 22 | 22 | 22 |
| CRPAZU090 | 941 | - | - | PDC-3 | OXA-396 | - | IMM, UZH | 0.5 | 0.25 | 0.25 | 0.008 | 0.008 | 0.125 | 34 | 34 | 30 |
| CRPAZU091 | 4936 | IMP-1 | - | PDC-35 | OXA-50 | OXA-10/PAC-1 | Haripur, Pakistan | **8** | 16 | 8 | 1 | 0.75 | 0.75 | 19 | 22 | 20 |
| CRPAZU092 | 4936 | IMP-1 | - | PDC-35 | OXA-50 | OXA-10/PAC-1 | Haripur, Pakistan | **8** | 16 | 64 | 2 | 0.75 | 0.75 | 19 | 21 | 20 |
| CRPAZU093 | 4936 | IMP-1 | - | PDC-35 | OXA-50 | OXA-10/PAC-1 | Haripur, Pakistan | **8** | 32 | 8 | 1.5 | 1 | 3 | 20 | 21 | 19 |
| CRPAZU094 | 1047 | - | GES-13 | PDC-12 | OXA-488 | OXA-10 | Haripur, Pakistan | 2 | 1 | 1 | 0.125 | 0.125 | 2 | 26 | 26 | 22 |
| CRPAZU 095 | 1047 | - | GES-13 | PDC-12 | OXA-488 | OXA-10 | Haripur, Pakistan | 1 | 1 | 1 | 0.125 | 0.125 | 0.25 | 24 | 25 | 24 |
| CRPAZU 096 | 235 | NDM-1/IMP-1 | - | PDC-35 | OXA-488 | OXA-101 | Haripur, Pakistan | 1 | 1 | 4 | 0.38 | 0.25 | 0.5 | 23 | 24 | 23 |
| CRPAZU 097 | 4936 | IMP-1 | - | PDC-35 | OXA-50 | OXA-10/PAC-1 | Haripur, Pakistan | **16** | 4 | 64 | 8 | 3 | 2 | 15 | 15 | 19 |
| CRPAZU 098 | 235 | NDM-1/IMP-1 | - | PDC-35 | OXA-488 | OXA-101 | Haripur, Pakistan | 2 | 1 | 16 | 0.25 | 0.19 | 0.25 | 24 | 24 | 24 |
| CRPAZU 099 | 4936 | IMP-1 | - | PDC-35 | OXA-50 | OXA-10/PAC-1 | Haripur, Pakistan | **8** | 32 | 64 | 3 | 0.75 | 1 | 18 | 20 | 20 |
| CRPAZU 100 | 654 | NDM-1 |  | PDC-3 | OXA-396 | - | Haripur, Pakistan | 2 | 2 | 8 | 1 | 0.75 | 0.75 | 22 | 23 | 22 |
| CRPAZU 101 | 395 | - | - | PDC-8 | OXA-905 | - | IMM, UZH | 0.25 | 0.5 | 1 | 0.125 | 0.94 | 0.25 | 27 | 26 | 24 |
| CRPAZU 102 | 235 | VIM-4 | - | PDC-35 | OXA-488 | OXA-35/CARB-2 | IMM, UZH | 1 | 0.25 | 1 | 0.125 | 0.125 | 0.38 | 27 | 29 | 26 |
| CRPAZU 103 | 317 | - | - | PDC-16 | OXA-488 | - | WRAIR collection | 0.5 | 0.25 | 1 | 0.25 | 0.125 | 0.38 | 24 | 26 | 24 |
| CRPAZU 104 | 5519 | - | - | PDC-35 | OXA-488 | - | WRAIR collection | 1 | 0.25 | 0.5 | 0.064 | 0.047 | 0.125 | 30 | 32 | 30 |
| CRPAZU 105 | 6220 | VIM-6 | - | PDC-1 | OXA-847 | OXA-10 | WRAIR collection | 2 | 2 | 4 | 1.5 | 0.5 | 2 | 19 | 21 | 18 |
| CRPAZU 106 | 6241 | - | PER-1 | PDC-62 | OXA-395 | OXA-2 | WRAIR collection | 2 | 2 | 4 | 0.38 | 0.25 | 2 | 22 | 24 | 17 |
| CRPAZU 107 | 6678 | - | - | PDC-35 | OXA-488 | OXA-2 | WRAIR collection | 2 | 2 | 2 | 0.19 | 0.19 | 1.5 | 24 | 25 | 21 |
| CRPAZU 108 | 8141 | - | PER-1 | PDC-33/62 | OXA-395 | OXA-2 | WRAIR collection | 2 | 2 | 4 | 0.38 | 0.19 | 3 | 23 | 25 | 19 |
| CRPAZU 109 | 8914 | - | - | PDC-3 | OXA-395 | OXA-10 | WRAIR collection | 1 | 0.5 | 2 | 0.25 | 0.19 | 0.25 | 26 | 27 | 27 |
| CRPAZU 110 | 23861 | KPC-2 | - | PDC-103 | OXA-50 | OXA-2/TEM-1A | WRAIR collection | 0.25 | 0.25 | 1 | 0.032 | 0.032 | 0.75 | 31 | 30 | 24 |
| CRPAZU 111 | 12914 | - | VEB-1 | PDC-11 | OXA-846 | OXA-10 | WRAIR collection | 1 | 2 | 4 | 0.75 | 0.75 | 1.5 | 22 | 22 | 20 |
| CRPAZU 112 | 20176 | VIM-11 | - | PDC-36 | OXA-395 | OXA-2 | WRAIR collection | 0.5 | 2 | 8 | 0.125 | 0.125 | 1 | 25 | 26 | 22 |

Resistant cefiderocol MICs are highlighted in bold.

**Table S2. Phenotypic features of *E.coli* overproducing PAC-1**

|  | **Minimal inhibitory concentration (mg/L)** | |
| --- | --- | --- |
| **Antibiotic** | **pTOPO-empty** | **pTOPO-*pac*-1** |
| **Piperacillin-Tazobactam** | 1 | 4 |
| **Ceftazidime** | 3 | 64 |
| **Ceftazidime/Avibactam** | 1 | 24 |
| **Ceftolozane/Tazobactam** | 1.5 | 16 |
| **Cefepim** | 0.38 | 4 |
| **Cefiderocol** | 0.016 | 0.5 |

**Table S3. Genes potentially associated with cefiderocol resistance in PAC-1-producing-*P. aeruginosa* isolates**

| **Gene** | **PAO1 Locus Tag** | **Genbank** | **Biological function** | **Amino acid alteration** |
| --- | --- | --- | --- | --- |
| *fstI* | PA4418 | AAG07806.1 | PBP3 | - |
| *exbB1* | PA0198 | AAG03587.1 | Inner membrane protein | - |
| *exbD1* | PA0199 | AAG03588.1 | Inner membrane protein | V97I |
| *tobB1* | PA5531 | AAG08916.1 | Inner membrane protein | Δ190-196, 218insP |
| *tonB2* | PA0197 | AAG03586.1 | Inner membrane protein | A171V, I166A |
| *tonB3* | PA0406 | AAG03795.1 | Inner membrane protein | V134A |
| *pirA* | PA0931 | AAG04320.1 | TBDR | S20N, T235I, A370N |
| *pirR* | PA0929 | AAG04318.1 | two component response regulator | G52A |
| *pirS* | PA0930 | AAG04319.1 | two component response sensor | R76Q, S125N, I180V, A328D |
| *pvdS* | PA2426 | AAG05814.1 | pyoverdine synthesis regulator | L180V, N182H |
| *fecA* | PA3901 | AAG07288.1 | TBDR | V95A, A113V, T288I, T349A, G358S, T359A, H363R, R570Q |
| *fecI* | PA3899 | AAG07286.1 | *fecA* regulator | - |
| *fecR* | PA3900 | AAG07287.1 | *fecI* regulator | H163Q, G191E, R207H, A230V, M250I, T294A |
| *piuB* | PA4513 | AAG07901.1 | *piuA* ortholog | N573D, V594A, N604H, A609V |
| *piuD* | - | XKH75407.1 | *piuA* ortholog | T411I |
| *fpvB* | PA4168 | AAG07555.1 | ferripyoverdine receptor | E262D |
| *oprD* | PA0958 | AAG04347.1 | outer membrane porin | loss |
| *ampC* | PA4110 | AAG07497.1 | beta-lactamase | G27D, A97V, T105A, V205L, G391A |
| *ampD* | PA4522 | AAG07910.1 | beta-lactamase regulator | G148A |
| *ampR* | PA4109 | AAG07496.1 | beta-lactamase regulator | G283W, M288R |

TBDR, tonB-dependent receptor; PBP, penicillin-binding protein
